# Accuracy of COVID-19–Like Illness Diagnoses in Electronic Health Record Data: Retrospective Cohort Study

**DOI:** 10.2196/39231

**Published:** 2023-01-17

**Authors:** Suchitra Rao, Catherine Bozio, Kristen Butterfield, Sue Reynolds, Sarah E Reese, Sarah Ball, Andrea Steffens, Maria Demarco, Charlene McEvoy, Mark Thompson, Elizabeth Rowley, Rachael M Porter, Rebecca V Fink, Stephanie A Irving, Allison Naleway

**Affiliations:** 1 Department of Pediatrics Hospital Medicine and Infectious Diseases University of Colorado School of Medicine Aurora, CO United States; 2 Centers for Disease Control and Prevention Atlanta, GA United States; 3 Westat Rockville, MD United States; 4 HealthPartners Institute Bloomington, MN United States; 5 Science Programs Department, Kaiser Permanente Center for Health Research Portland, OR United States

**Keywords:** COVID-19, COVID-like illness, COVID-19 case definition, sensitivity, specificity, positive predictive value, negative predictive value

## Abstract

**Background:**

Electronic health record (EHR) data provide a unique opportunity to study the epidemiology of COVID-19, clinical outcomes of the infection, comparative effectiveness of therapies, and vaccine effectiveness but require a well-defined computable phenotype of COVID-19–like illness (CLI).

**Objective:**

The objective of this study was to evaluate the performance of pathogen-specific and other acute respiratory illness (ARI) International Statistical Classification of Diseases-9 and -10 codes in identifying COVID-19 cases in emergency department (ED) or urgent care (UC) and inpatient settings.

**Methods:**

We conducted a retrospective observational cohort study using EHR, claims, and laboratory information system data of ED or UC and inpatient encounters from 4 health systems in the United States. Patients who were aged ≥18 years, had an ED or UC or inpatient encounter for an ARI, and underwent a SARS-CoV-2 polymerase chain reaction test between March 1, 2020, and March 31, 2021, were included. We evaluated various CLI definitions using combinations of International Statistical Classification of Diseases-10 codes as follows: COVID-19–specific codes; CLI definition used in VISION network studies; ARI signs, symptoms, and diagnosis codes only; signs and symptoms of ARI only; and random forest model definitions. We evaluated the sensitivity, specificity, positive predictive value, and negative predictive value of each CLI definition using a positive SARS-CoV-2 polymerase chain reaction test as the reference standard. We evaluated the performance of each CLI definition for distinct hospitalization and ED or UC cohorts.

**Results:**

Among 90,952 hospitalizations and 137,067 ED or UC visits, 5627 (6.19%) and 9866 (7.20%) were positive for SARS-CoV-2, respectively. COVID-19–specific codes had high sensitivity (91.6%) and specificity (99.6%) in identifying patients with SARS-CoV-2 positivity among hospitalized patients. The VISION CLI definition maintained high sensitivity (95.8%) but lowered specificity (45.5%). By contrast, signs and symptoms of ARI had low sensitivity and positive predictive value (28.9% and 11.8%, respectively) but higher specificity and negative predictive value (85.3% and 94.7%, respectively). ARI diagnoses, signs, and symptoms alone had low predictive performance. All CLI definitions had lower sensitivity for ED or UC encounters. Random forest approaches identified distinct CLI definitions with high performance for hospital encounters and moderate performance for ED or UC encounters.

**Conclusions:**

COVID-19–specific codes have high sensitivity and specificity in identifying adults with positive SARS-CoV-2 test results. Separate combinations of COVID-19-specific codes and ARI codes enhance the utility of CLI definitions in studies using EHR data in hospital and ED or UC settings.

## Introduction

Electronic health record (EHR) data provide a unique opportunity to study the epidemiology of COVID-19, clinical outcomes of infection, comparative effectiveness of therapies, and vaccine effectiveness (VE). For example, COVID-19 vaccines are highly effective against SARS-CoV-2 infection [1-3], but emerging evidence regarding waning immunity [4-7] and the emergence of novel variants [8,9] requires robust and ongoing evaluations of VE [4,7,10] against hospitalizations and other outcomes such as ambulatory, emergency department (ED), and urgent care (UC) visits. A standardized and reliable definition of COVID-19–like illness (CLI) would enhance the quality of real-world effectiveness studies using EHR data sources. However, computable phenotypes for CLI definitions require further definition and evaluation.

A diagnosis code for COVID-19 (*International Statistical Classification of Diseases, Tenth Revision, Clinical Modification* [*ICD-10-CM*] code U07.1) was introduced in the United States on April 1, 2020 [11], but the reliability of this and other COVID-19–specific codes (eg, J12.82, pneumonia due to COVID-19) in identifying CLI has not been widely studied. Concurrently, it is unknown which existing acute respiratory illness (ARI) codes drawn from studies conducted before the pandemic [12,13] and now used in COVID-19 VE studies [14] have sufficient sensitivity and specificity to identify laboratory-confirmed cases and whether these definitions will need to differ across different health care settings and age groups. Assessing the accuracy of diagnostic codes and computable phenotypes is essential for ensuring the validity and reliability of these EHR data sources. The use of laboratory results as a reference standard has been used as a standard approach to evaluate the accuracy of ICD codes; however, studies have demonstrated poor performance for other infectious diseases, including influenza [15-18]. Therefore, the objectives of this study were to evaluate the sensitivity, specificity, positive predictive value (PPV), and negative predictive value (NPV) of different combinations of ICD codes in identifying polymerase chain reaction (PCR)–confirmed SARS-CoV-2 infection in adult patients in ED or UC and hospitalized settings. Next, we sought to determine which combination of diagnostic codes achieved a CLI definition with enhanced sensitivity and specificity that could be utilized for future epidemiological and VE studies using EHR data.

## Methods

### Study Design and Population or Data Source

We conducted a retrospective analysis using EHR, claims, and laboratory information system data from health systems within the VISION Network: HealthPartners (Minnesota and Wisconsin), Kaiser Permanente Northwest (Oregon and Washington), University of California Health, and University of Colorado Health. The health systems in these analyses represent 87 hospitals, 85 EDs, and 83 UC centers. Our patient cohort included persons who were aged ≥18 years and had ≥1 ambulatory visit within the 4 health systems in the 12 months before September 1, 2019 (defined as the look-back period). For Kaiser Permanente Northwest and HealthPartners, active membership in the health system was also required during the period from the look back to the end of the study (March 31, 2021), disenrollment, or death, whichever occurred first. Data on encounters were collected if the encounter had an ARI diagnosis or a respiratory virus test performed. This analysis included all cohort members who underwent a SARS-CoV-2 PCR test (including symptomatic and asymptomatic patients) from 14 days before through 72 hours after an inpatient, UC, or ED encounter for CLI (definition is provided in the subsequent section) between March 1, 2020, and March 31, 2021. Hospitalizations were included if the length of stay was ≥24 hours. Multiple visits per patient were permitted in the analyses and could be included in both the inpatient and outpatient cohorts. We excluded patients with inconclusive SARS-CoV-2 PCR test results.

### Data Collection and Variable Selection

We defined CLIs using ICD, ninth and tenth revision diagnoses, and sign and symptom codes from hospital discharge and ED or UC encounters, based on previous studies of COVID-19 [19-21]. The VISION CLI case definition required 1 or more of the following diagnoses: COVID-19, COVID-19 pneumonia, influenza pneumonia, other viral pneumonia, bacterial pneumonia, influenza disease, acute respiratory distress syndrome, chronic obstructive pulmonary disease (COPD) exacerbation, asthma exacerbation, respiratory failure, other acute lower respiratory tract infections, acute upper respiratory tract infections, signs and symptoms of ARI such as cough and tachypnea, and signs and symptoms of certain acute nonrespiratory conditions [14,22,23] (Multimedia Appendix 1). Codes were included if they were a principal or secondary diagnosis during the health care encounter.

Data from hospital readmissions within 30 days of discharge, repeat ED encounters within 24 hours, or repeat UC encounters within 24 hours were combined and analyzed as single medical encounters within each setting. Encounters with a SARS-CoV-2 PCR test occurring ≤14 days before <72 hours after a hospital admission or an ED or a UC encounter were selected as the reference standard to represent COVID-19–associated hospitalizations and encounters.

### CLI Definitions

We assessed the performance of various CLI definitions. The first definition used only COVID-19 ICD-10 codes (U07.1, B34.2, J12.81, and J12.82). The second was the VISION CLI definition. We used a third definition of CLI using signs and symptom ICD codes of ARI and a fourth definition of ARI signs, symptoms, and diagnoses alone. The specific ICD codes for the definitions above are listed in Multimedia Appendix 1.

### Statistical Analyses

The sociodemographic and clinical characteristics of the study population were summarized by test result using proportions for categorical variables. To determine the performance of each CLI definition, we evaluated the sensitivity, specificity, PPV, and NPV against the reference standard (SARS-CoV-2 PCR positive test result). We evaluated the performance of each CLI definition for distinct hospitalization and ED or UC cohorts.

We used a random forest classification method to identify the groups of ICD-9 and -10 codes with the highest sensitivity and specificity for identifying COVID-19 (defined as a positive SARS-CoV-2 PCR test result) [24]. Random forest classification is a type of machine-learning algorithm used to predict binary outcomes by averaging predictions from a set of nonparametric recursive decision trees. The method can be used as an alternative to logistic regression when sample sizes are very large and complex interactions exist among many independent covariates [25]. A total of 2 random forest models were developed for the inpatient and ED or UC cohorts separately. The first model contained all codes in the VISION CLI definition. The second model contained CLI groups included in Multimedia Appendix 1 but excluded COVID-19 and COVID-19 pneumonia codes.

For each model, the cohort data were randomly split into a training set for model fitting and a test set for performance evaluation. The training set comprised 80% of the full cohort, and the test cohort comprised the remaining 20%. The low SARS-CoV-2 positivity rate in our cohort created a class imbalance between the majority class (observations without SARS-CoV-2 positivity) and minority class (SARS-CoV-2 positivity by PCR). To account for this imbalance, we performed random undersampling on the majority class of the training data set to balance the 2 groups, thereby generating a 1:1 class ratio for modeling. Additional covariates in the models were age, sex, race or ethnicity, site or region, and any underlying medical condition associated with the encounter of interest. Hyperparameters were tuned as follows: 250 to 500 trees were included per model, tree depth was between 3 and 4, between 4 and 6 features randomly selected per tree, and 75% of the data were used for bagging per tree. We calculated the sensitivity, specificity, PPV, NPV, area under the receiver operating characteristic curve (AUROC), 95% CI to evaluate the model performance. The AUROC ranges from 0.5 to 1, and the higher the value, the better the model is in distinguishing the positive SARS-CoV-2 results from negative SARS-CoV-2 results. Variable importance plots, based on the mean decrease in accuracy and mean decrease in the Gini coefficient, were assessed to determine the top ICD codes for the prediction of SARS-CoV-2 positivity.

### Sensitivity Analyses

We conducted a sensitivity analysis of hospitalized and ED or UC patients from HealthPartners using the available testing indication data. These data differentiated whether the person was symptomatic or asymptomatic for COVID-19 based on ordering provider assessment when the specimen was collected for SARS-CoV-2 testing at any medical facility within the HealthPartners system. Patients identified as symptomatic or asymptomatic with positive SARS-CoV-2 PCR results were used as the reference standards for 2 separate analyses.

### Ethics Approval

This study was reviewed and approved by the institutional review board of Westat, Inc (45 code of federal regulations part 46; 21 Code of federal regulations part 56).

## Results

### Participant Enrollment Description

Of the 118,740 hospitalizations in the cohort, 94,643 (79.71%) had SARS-CoV-2 testing performed within the study period. Among the 24,097 (20.29%) patients who did not undergo testing within the study period, 8.10% (n=1952) were admitted from another acute inpatient setting. Excluding pediatric hospitalizations, a total of 90,952 (76.6%) adult hospitalizations were included in the analyses. Among the 90,952 hospital encounters, 36,877 (40.55%) had testing performed within the 14 days before admission. Of the 207,056 ED or UC encounters in the cohort, 149,848 (72.37%) had SARS-CoV-2 testing performed within the specified time frame. Excluding pediatric encounters, 137,067 (66.2%) ED or UC encounters were included in the analyses. Of these, 5627 (4.11%) hospitalizations and 9866 (7.2%) ED or UC encounters were associated with positive SARS-CoV-2 results.

### Participant Characteristics

The sociodemographic and clinical characteristics of the patients in each of the 2 cohorts are summarized in Table 1.

In sum, in the hospitalized cohort, 55.3% (50,326/90,952) of the patients were female, 65.8% (59,830/90,952) of the patients were of White non-Hispanic race or ethnicity, and 45.7% of the patients were aged >65 years. Most patients (80.7%, 73,379/90,952) had at least one underlying medical condition, and 47.4% (43,118/90,952) had public insurance. In the ED or UC cohort, 59.7% (81,852/137,067) of the patients were female, 70% (95,910/137,067) of the patients were White and non-Hispanic, 28.3% (38,855/137,067) of the patients were aged >65 years, 65% (89.068/137,067) of the patients had at least one underlying medical condition, and 33.4% (45,814/137,067) of the patients had public insurance (Table 2).

**Table 1:** Demographic characteristics of patients hospitalized and tested for SARS-CoV-2.

Characteristics		All SARS-CoV-2–tested hospitalizations (n=90,952), n (%)	SARS-CoV-2–positive hospitalizations (n=5627), n (%)	SARS-CoV-2–negative hospitalizations (n=85,325), n (%)
**Sex**				
	Male	40,621 (44.7)	2794 (49.7)	37,827 (44.3)
	Female	50,326 (55.3)	2832 (50.3)	47,494 (55.7)
	Other or unknown	5 (0)	1 (0)	4 (0)
**Age (years)**				
	18-24	3563 (3.9)	144 (2.6)	3419 (4)
	25-49	23,139 (25.4)	1121 (19.9)	22,018 (25.8)
	50-64	22,690 (24.9)	1587 (28.2)	21,103 (24.7)
	>65	41,560 (45.7)	2775 (49.3)	38,785 (45.5)
**Race or ethnicity**				
	White and non-Hispanic	59,830 (65.8)	2878 (51.1)	56,952 (66.7)
	Black and non-Hispanic	7216 (7.9)	491 (8.7)	6725 (7.9)
	Asian and non-Hispanic	5204 (5.7)	337 (6)	4867 (5.7)
	Hispanic or Latinx	13,498 (14.8)	1581 (28.1)	11,917 (14)
	Other	2456 (2.7)	179 (3.2)	2277 (2.7)
	Unknown	2748 (3)	161 (2.9)	2587 (3)
**Study site**				
	HealthPartners	5376 (5.9)	445 (7.9)	4931 (5.8)
	Kaiser Permanente Northwest	14,299 (15.7)	812 (14.4)	13,487 (15.8)
	University of California Health	36,827 (40.4)	1778 (31.6)	35,049 (41.1)
	University of Colorado	34,450 (37.9)	2592 (46.1)	31,858 (37.3)
**ICU^a^ admission**				
	Yes	15,468 (17)	1350 (24)	14,118 (16.5)
	No	75,484 (83)	4277 (76)	71,207 (83.5)
**Underlying conditions**				
	Yes	73,379 (80.7)	4733 (84.1)	68,646 (80.5)
	No	17,573 (19.3)	894 (15.9)	16,679 (19.5)
**Primary insurance type**				
	Medicare	27,782 (30.5)	1780 (31.6)	26,002 (30.5)
	Medicaid	15,336 (16.9)	1005 (17.9)	14,331 (16.8)
	Private	15,251 (16.8)	1083 (19.2)	14,168 (16.6)
	Other	15,967 (17.6)	769 (13.7)	15,198 (17.8)
	Unknown	16,616 (18.3)	990 (17.6)	15,626 (18.3)

^a^ICU: intensive care unit.

**Table 2:** Demographic characteristics of patients evaluated in an emergency department (ED) or urgent care (UC) setting and tested for SARS-CoV-2.

Characteristic			All SARS-CoV-2–tested ED or UC visits (n=137,067), n (%)		Positive ED or UC visits (n=9866), n (%)		Negative ED or UC visits (n=127,201), n (%)
**Sex**							
	Male	55,188 (40.3)		4108 (41.6)		51,080 (40.2)	
	Female	81,852 (59.7)		5757 (58.4)		76,095 (59.8)	
	Other or unknown	27 (0)		1 (0)		26 (0)	
**Age (years)**							
	18-24	9838 (7.2)		848 (8.6)		8990 (7.1)	
	25-49	49,929 (36.4)		4133 (41.9)		45,796 (36)	
	50-64	38,445 (28)		2780 (28.2)		35,665 (28)	
	>65	38,855 (28.3)		2105 (21.3)		36,750 (28.9)	
**Race or ethnicity**							
	White and non-Hispanic	95,910 (70)		5270 (53.4)		90,640 (71.3)	
	Black and non-Hispanic	8057 (5.9)		900 (9.1)		7157 (5.6)	
	Asian and non-Hispanic	7753 (5.7)		618 (6.3)		7135 (5.6)	
	Hispanic or Latinx	15,514 (11.3)		2204 (22.3)		13,310 (10.5)	
	Other	4342 (3.2)		412 (4.2)		3930 (3.1)	
	Unknown	5491 (4)		462 (4.7)		5029 (4)	
**Study site**							
	HealthPartners	34,232 (25)		2888 (29.3)		31,344 (24.7)	
	Kaiser Permanente Northwest	47,087 (34.4)		3049 (30.9)		44,038 (34.6)	
	University of California Health	45,149 (32.9)		2702 (27.4)		42,447 (33.4)	
	University of Colorado	10,599 (7.7)		1227 (12.4)		9372 (7.4)	
**Underlying conditions**							
	Yes	89,068 (65)		5955 (60.4)		83,113 (65.3)	
	No	47,999 (35)		3911 (39.6)		44,088 (34.7)	
**Insurance type**							
	Medicare	30,133 (22)		1555 (15.8)		28,578 (22.5)	
	Medicaid	15,681 (11.4)		1597 (16.2)		14,084 (11.1)	
	Private	54,730 (39.9)		4351 (44.1)		50,379 (39.6)	
	Other	30,253 (22.1)		1729 (17.5)		28,524 (22.4)	
	Unknown	6270 (4.6)		634 (6.4)		5636 (4.4)	

### Model Performance

Table 3 summarizes the sensitivity, specificity, PPV, and NPV for each CLI definition among hospitalizations. Among CLI hospitalizations in adults, COVID-19–specific codes had the highest sensitivity (91.6%) and specificity (99.6%) in identifying patients with SARS-CoV-2 PCR positivity.

Using the VISION CLI definition, the sensitivity remained high (95.8%), but the specificity was considerably lower (45.5%). By contrast, the signs and symptoms of ARI had low sensitivity and PPV (28.9% and 11.8%, respectively) but higher specificity and NPV (85.3% and 94.7%, respectively). Using ARI signs, symptoms, and diagnoses alone, sensitivity and specificity were 76.4% and 60.6%, respectively.

The evaluation of individual codes among the hospitalized patients confirmed that the “COVID-19, virus identified” ICD-10 code (U07.1) was associated with the highest odds of having a SARS-CoV-2 test result, followed by the COVID-19 pneumonia codes (pneumonia due to SARS-associated coronavirus and pneumonia due to COVID-19).

As shown in Table 4, all CLI definitions had a lowered sensitivity for ED or UC encounters using the reference standard of SARS-CoV-2 PCR positivity. COVID-19–specific codes had a sensitivity of 32.8% but retained high specificity (99.6%), whereas the VISION CLI definition also had lower sensitivity (49.1%) but improved specificity (74.2%). A CLI definition using signs and symptoms alone did not have improved performance in the ED or UC cohort compared with the hospitalized cohort (sensitivity 22.3%; specificity 87.8%).

**Table 3:** Sensitivity, specificity, positive predictive value (PPV), and negative predictive value (NPV) of various definitions of COVID-19–like illnesses (CLIs) using International Statistical Classification of Diseases (ICD)-10 codes in identifying hospitalized adult patients with SARS-CoV-2 infection using polymerase chain reaction (PCR) detection as the reference standard (n=87,771).

CLI definition^a^ using PCR results as the gold standard	Sensitivity, % (95% CI)	Specificity, % (95% CI)	PPV, % (95% CI)	NPV, % (95% CI)
COVID-19–specific codes	91.6 (90.9-92.3)	99.6 (99.5-99.6)	93.4 (92.7-94.0)	99.4 (99.4-99.5)
VISION CLI	95.8 (95.3-96.4)	45.5 (45.2-45.9)	10.7 (10.4-10.9)	99.4 (99.3-99.5)
Signs and symptoms of ARI^b^	28.9 (27.7-30.1)	85.3 (85.1-85.6)	11.8 (11.3-12.4)	94.7 (94.5-94.8)
ARI diagnoses alone	76.4 (75.3-77.5)	60.6 (60.3-60.9)	11.3 (11.0-11.7)	97.5 (97.4-97.6)
Random forest analysis: highest predictive codes^c^	93.6 (92.9-94.2)	83.5 (83.2-83.8)	27.8 (27.1-28.4)	99.5 (99.4-99.5)
Random forest analysis: VISION CLI excluding COVID-19–specific codes	61.7 (58.7-64.5)	81.9 (81.4-82.5)	18.9 (17.6-20.2)	96.9 (96.6-97.2)

^a^CLI definitions are outlined in Multimedia Appendix 1.

^b^ARI: acute respiratory illness.

^c^Codes with the highest predictivity based on Gini and accuracy measures using random forest analyses included COVID-19, virus identified (U07.1), acute respiratory failure (ICD-10 code J96.0 and ICD-9 code 518.81), pneumonia due to COVID-19 (J12.82), hypoxemia (ICD-10 code R09.02 and ICD-9 code 799.02), asphyxia or hypoxemia (R09.0), other bacterial pneumonia (ICD-10 code J15 and ICD-9 code 482), and chronic obstructive pulmonary disease with acute lower respiratory tract infection (ICD-10 code J44.0 and ICD-9 code 419.22).

**Table 4:** Sensitivity, specificity, positive predictive value (PPV), and negative predictive value (NPV) of various COVID-19–like illness (CLI) definitions using International Statistical Classification of Diseases (ICD)-10 codes in identifying the patients evaluated in an emergency department (ED) or urgent care (UC) setting with SARS-CoV-2 infection using polymerase chain reaction (PCR) detection as the reference standard.

CLI definition^a^ using PCR results as the gold standard	Sensitivity, % (95% CI)	Specificity, % (95% CI)	PPV, % (95% CI)	NPV, % (95% CI)
COVID-19–specific codes	32.8 (31.9-33.8)	99.6 (99.5-99.6)	85.8 (84.7-86.9)	95 (94.9-95.2)
VISION CLI	49.1 (48.1-50.1)	74.2 (74.0-74.4)	12.9 (12.5-13.2)	94.9 (94.8-95.1)
Signs and symptoms of ARI^b^	22.3 (21.5-23.1)	87.8 (87.6-87.9)	12.4 (11.9-12.9)	93.6 (93.4-93.7)
ARI diagnoses alone	34.7 (33.8-35.7)	78.4 (78.2-78.6)	11.1 (10.7-11.4)	93.9 (93.8-94.1)
Random forest analysis: highest predictive codes^c^	44.6 (43.6-45.6)	89.4 (89.2-89.5)	24.5 (23.9-25.2)	95.4 (95.3-95.5)
Random forest analysis: VISION CLI excluding COVID-19–specific codes	46.5 (44.3-48.7)	77.4 (76.9-77.9)	13.7 (12.9-14.6)	94.9 (94.6-95.2)

^a^CLI definitions are outlined in Multimedia Appendix 1.

^b^ARI: acute respiratory illness.

^c^Codes with the highest predictivity based on Gini and accuracy measures using random forest analyses included COVID-19, virus identified (U07.1), cough (ICD-10 code R05 and ICD-9 code 786.2), disturbance of smell and taste (R43), fever (R50), fever, unspecified (ICD-10 code R50.9 and ICD-9 code 780.6), pneumonia, unspecified organism (ICD-10 code J18 and ICD-9 code 486), pneumonia due to COVID-19 (J12.82), and myalgia (ICD-10 code M79.1 and ICD-9 code 729.1).

### Random Forest Analyses

The split cohort resulted in 69,933 (80%) and 17,838 (20%) patients in the training and test hospitalization data sets, respectively, and 109,591 (80%) and 27,476 (20%) patients in the ED and UC data sets, respectively. Observations with missing diagnostic codes were removed from the data sets (3181 hospital and 0 ED or UC were excluded). After performing random undersampling, the balanced training set consisted of 4417 SARS-CoV-2–negative and 4417 SARS-CoV-2–positive hospitalized events. Codes yielding the highest predictive performance included COVID-19, pneumonia due to COVID-19, asphyxia and hypoxemia, acute respiratory failure, hypoxemia, other bacterial pneumonia, and COPD with acute lower respiratory tract infection; using only these diagnostic codes led to a sensitivity of 93.6%, specificity of 83.5%, PPV of 27.8%, and NPV of 99.5%, with an AUROC of 0.89 (Table 4).

For the ED or UC cohort, the balanced data set included 7891 positive and 7891 negative observations. The top predictive codes included COVID-19, cough, disturbance of smell and taste, fever, fever (unspecified), pneumonia (unspecified organism), pneumonia due to COVID-19, and myalgia. These codes alone had a sensitivity of 44.6%, a specificity of 89.4%, a PPV of 24.5%, an NPV of 95.4%, and an AUROC of 0.67. Excluding the COVID-19–specific diagnosis codes lowered the sensitivity and specificity, as outlined in Table 4.

### Sensitivity Analyses

Using the data from 17.5% (343/1961) symptomatic patients among SARS-CoV-2–positive hospitalizations from HealthPartners as the reference standard, sensitivity and specificity for COVID-19–specific ICD-10 codes remained high (100% and 97.2%, respectively), but for definitions using ARI codes, there was a loss of specificity (6.2% for the VISION CLI definition, 16.6% for ARI diagnoses alone, and 38.8% for signs and symptoms alone; Table 5).

Restricting our analyses to 78 (3.7%) asymptomatic patients from the 2109 SARS-CoV-2–associated hospitalizations with positive test results, the sensitivity (97.5%) and specificity (99.2%) for COVID-19–related ICD-10 codes remained high. Sensitivity (97.5%) was also high for the full VISION CLI definition; however, specificity (36.0%) dropped markedly.

**Table 5:** Sensitivity, specificity, positive predictive value (PPV), and negative predictive value (NPV) for the various definitions of COVID-19–like illnesses (CLIs) using the reference standard of symptomatic and asymptomatic^a^ SARS-CoV-2–positive hospitalizations (HealthPartners data only, n=1961)^b^.

		Sensitivity, % (95% CI)	Specificity, % (95% CI)	PPV, % (95% CI)	NPV, % (95% CI)
**Symptomatic hospitalized patients with SARS-CoV-2–positive testing**					
	COVID-19–specific codes	100	97.2 (94.4-98.0)	88.4 (85.2-91.6)	100
	VISION CLI definition	100	6.2 (5.1-7.4)	18.4 (16.7-20.2)	100
	Signs and symptoms of ARI^c^	75.8 (71.3-80.3)	38.8 (36.4- 41.1)	20.8 (18.5-23.0)	88.3 (86.0-90.7)
	ARI diagnoses alone	96.8 (94.9-98.7)	16.6 (14.8-18.4)	19.8 (17.9-21.7)	96.1 (93.8-98.4)
	Random forest analysis: highest predictive codes^d^	100 (98.9-100)	66.3 (64.0-68.6)	38.6 (35.4-41.9)	100 (99.7-100)
	Random forest analysis: VISION CLI excluding COVID-19–specific codes	67.6 (55.2-78.5)	81.8 (77.1-85.8)	44.2 (34.5-54.3)	92.2 (88.4-95.0)
**Asymptomatic hospitalized patients with SARS**-**CoV-2–positive testing**					
	COVID-19–specific codes	97.5 (94.0-100)	99.2 (98.9-99.6)	76.2 (67.9-84.5)	99.9 (99.9-100)
	VISION CLI	98.7 (96.3-100)	36 (34.4-37.7)	3.7 (2.9-4.5)	99.9 (99.7-100)
	Signs and symptoms of ARI	50.6 (39.6-61.7)	81.4 (80.0-82.7)	6.3 (4.4-8.2)	98.5 (98.1-99.0)
	ARI diagnoses alone	70.9 (60.9-80.9)	60.8 (59.1-62.5)	4.3 (3.2-5.4)	98.8 (98.3-99.3)
	Random forest analysis: highest predictive codes^e^	98.7 (93.1-100)	80.0 (78.6-81.4)	11.0 (8.8-13.5)	100 (99.8-100)
	Random forest analysis: VISION CLI excluding COVID-19–specific codes	42.9 (17.7-71.1)	93.3 (91.1-95.1)	12.2 (4.6-24.8)	98.7 (97.4-99.4)

^a^Symptomatic and asymptomatic designations were based on test indication data completed by ordering provider at time of test order.

^b^CLI definitions are outlined in Multimedia Appendix 1.

^c^ARI: acute respiratory illness.

^d^COVID-19, virus identified, acute respiratory failure, pneumonia due to COVID-2019, hypoxemia, asphyxia and hypoxemia, and other bacterial pneumonia.

^e^COVID-19, virus identified, acute respiratory failure, asphyxia and hypoxemia, hypoxemia, cough, pneumonia, unspecified organism, and altered level of consciousness or altered mental status.

## Discussion

### Principal Findings

In this multicenter cohort study of adults undergoing SARS-CoV-2 testing in 4 large, integrated health systems, we found high sensitivity of our existing VISION CLI definition for hospitalized patients, which uses ICD-10 diagnoses and sign and symptom codes that have been associated with COVID-19 in previous studies. However, this definition had a lower sensitivity for ED or UC encounters. Signs and symptoms alone had low sensitivity but higher specificity than VISION CLI for both hospitalization and ED or UC encounters. COVID-19 codes alone were not able to adequately differentiate symptomatic from asymptomatic hospitalizations, given the similar performance characteristics between the 2 groups. Using random forest classification methods, the combination of COVID-19, COVID-19 pneumonia, bacterial pneumonia, acute respiratory failure, COPD with acute lower respiratory tract infection, hypoxemia, and asphyxia or hypoxemia diagnoses codes had high sensitivity and specificity in identifying a SARS-CoV-2–positive hospitalization. A different combination of codes (COVID-19, cough, disturbance of smell and taste, fever, fever [unspecified], pneumonia unspecified organism, pneumonia due to COVID-19, and myalgia) were used for similar model prediction for ED or UC encounters, but the overall performance remained lower, as compared with the hospitalization findings. These analyses enhance our understanding of the use of ICD-10 codes to generate specific computable phenotypes for CLI that can be used in future EHR-based epidemiological studies of COVID-19 illness and VE studies.

Calculating VE against important real-world outcomes using EHR data requires the ability to identify CLI-associated hospitalizations and cannot solely rely on SARS-CoV-2 test results, given that many medical facilities practice universal testing of patients and may include patients with asymptomatic COVID-19 infection among patients being hospitalized for unrelated reasons. Conversely, diagnostic codes alone have been shown to be inaccurate for case identification and classification in epidemiological surveillance studies [26,27]. This study provided several important insights into the use of EHR data to define CLIs. First, we validated our prior approach to identify CLIs, which is being used in studies using the VISION network to estimate VE against COVID-19–associated hospitalizations and ED or UC encounters [2,12,18,19]. Second, we generated a more refined CLI definition using random forest methods with high sensitivity and specificity for future studies. Third, we evaluated the accuracy of COVID-19–specific codes in the identification of SARS-CoV-2 infection in different health care settings.

A new ICD-10-CM code for COVID-19 (U07.1) was introduced on April 1, 2020, to facilitate billing and case monitoring. Hospitals rapidly began using the new ICD-10-CM code for COVID-19 (U07.1) within 2 weeks of its release [28]. Our data reflect the rapid uptake of these diagnostic codes among the health systems in our study, with high sensitivity and specificity for the U-code COVID-19, virus identified (U07.1), and B-code coronavirus infection, unspecified. A study using the Premier Healthcare Database (an administrative all-payer repository that covers approximately 20% of all US hospitalizations from 48 states) conducted between January 1, 2020, and May 31, 2020, found similar sensitivity, specificity, PPV, and NPV for the ICD-10 code U07.1 among hospitalized adults, using SARS-CoV-2 PCR test results as the reference standard [28]. A similar approach was taken by investigators at the Yale and Mayo Clinic but yielded contrary findings. They reported a higher misclassification by COVID-19 diagnostic codes, with a sensitivity of 83.3% and PPV of 68.8% of a CLI diagnosis code in the medical record among patient records with a documented positive SARS-CoV-2 test [29]. This and other studies have cautioned against the sole reliance of these codes to identify SARS-CoV-2 infections, demonstrating that the sensitivity may decrease over time [29-31] and may also be lower among younger age groups [29].

To our knowledge, our analyses represent the first evaluation of EHR-based CLI definitions in the ED and UC settings. We found that these definitions had lower sensitivity than the inpatient setting, even when limited to COVID-19–specific codes (the sensitivity was 91.6% for the inpatient cohort vs 32.8% for the outpatient cohort). This finding may result from the coding of these visits occurring before SARS-CoV-2 test results are available or coding based on test results outside the health system. Therefore, alternative definitions may need to be considered in these settings. Random forest analyses selecting the highest predictive codes demonstrated improved specificity from the VISION CLI diagnosis by approximately 15% in the ED or UC setting, suggesting that acute respiratory symptoms and signs, coupled with pneumonia and COVID-19 codes, may better define positivity in this cohort, given that the proportion of patients with milder presentations is higher than that of hospitalized patients.

To further refine our CLI diagnoses for potential use in future studies, we used a random forest approach to identify a group of ICD-10 codes that maximized the sensitivity and specificity for identifying COVID-19 infection. This approach has several advantages in studies using EHR data because it can handle large data sets efficiently, uses nonparametric statistical procedures, focuses on optimizing accuracy in predicting outcomes, and identifies and ranks variables that are important in predicting outcomes while accounting for all interaction effects [32]. Using this approach, we identified the codes that had the highest predictive accuracy at identifying SARS-CoV-2 infection. These ICD-10 codes included viral pneumonia and respiratory failure, which have been shown in other studies to be strongly associated with COVID-19 infection [33]. Fever, cough, hypoxemia, and disturbance of smell and taste were the sign and symptom codes with the highest predictive accuracy in the ED or UC cohort, which has also been observed in other studies across different age groups [34].

### Limitations

The strengths of our study include a large sample size from a geographically diverse sample, with reliable testing data available at each site. However, our study had several limitations worth noting. The ideal reference standard would be hospitalizations and ED or UC visits attributed to SARS-CoV-2 infection, but we did not have reliable testing indication data for all sites and, therefore, were restricted to a sensitivity analysis at 1 site. Next, testing performed within the network partners’ medical facilities was captured in the EHR data; however, if testing was performed outside of the partners’ medical facilities and yielded positive results, outcome misclassification is possible. Next, collinearity between diagnostic codes and test positivity may exist for hospitalized patients at some sites, whereby a positive test may trigger a COVID-19–specific discharge code. Finally, we did not evaluate the performance of primary versus secondary diagnoses or evaluate changes in performance over time, which will be an important focus of future research.

### Conclusions

CLI definitions that maximize sensitivity and specificity in this study could be applied to COVID-19–related studies in which universal SARS-CoV-2 testing may not be available or in other EHR-based analyses with limited or no access to laboratory data. These findings can help refine specific computable phenotypes for CLIs that can be used in future epidemiological studies of COVID-19 illness and studies evaluating the effectiveness of COVID-19 vaccines against hospitalization and other clinical end points.

## Appendix

Multimedia Appendix 1 International Statistical Classification of Diseases (ICD)-9 and -10 codes for conditions, signs, and symptoms used for definitions of COVID-19–like illnesses (CLIs).

## Abbreviations

ARI: acute respiratory illness
AUROC: area under the receiver operating characteristic curve
CLI: COVID-19–like illness
COPD: chronic obstructive pulmonary disease
ED: emergency department
EHR: electronic health record
ICD: International Classification of Diseases
NPV: negative predictive value
PCR: polymerase chain reaction
PPV: positive predictive value
UC: urgent care
VE: vaccine effectiveness
