# Supplementary material for: Accuracy of COVID-19–Like Illness Diagnoses in Electronic Health Record Data: Retrospective Cohort Study
Source: JMIR Form Res. 2023 Jan 17;7:e39231. doi: 10.2196/39231 (PMC9848441; doi:10.2196/39231)
Supplement: Multimedia Appendix 1 [file formative_v7i1e39231_app1.docx]

Multimedia Appendix 1

**Table S1.** International Statistical Classification of Diseases (ICD)-9 and -10 codes for conditions, signs, and symptoms used for definitions of COVID-19–like illnesses (CLIs).

| Disease conditions | | ICD-10 codes | ICD-9 codes | COVID-19–specific codes | Codes used in VISION-CLI definition^a^ | Codes used in signs and symptoms of ARI^b^ definition | Codes used in ARI signs, symptoms, and diagnoses definition |
| --- | --- | --- | --- | --- | --- | --- | --- |
| **COVID-19** | | | | | | | |
|  | COVID-19, virus identified | U07.1 | N/A^c^ | ✓^d^ | ✓ |  |  |
|  | COVID-19, virus not identified | U07.2 | N/A | ✓ | ✓ |  |  |
|  | COVID-19 infection, unspecified | B34.2 | N/A | ✓ | ✓ |  |  |
| **COVID-19 pneumonia** | | | | | | | |
|  | Pneumonia due to SARS^e^-associated coronavirus | J12.81 | N/A | ✓ | ✓ |  |  |
|  | Pneumonia due to COVID-19 | J12.82 | N/A | ✓ | ✓ |  |  |
| **Influenza pneumonia** | | | | | | | |
|  | Influenza due to the identified novel influenza A virus with pneumonia | J09.X1 | 488.81 |  | ✓ |  | ✓ |
|  | Influenza due to other identified influenza virus with pneumonia | J10.0 | N/A |  | ✓ |  | ✓ |
|  | Influenza due to other identified influenza virus with unspecified type of pneumonia | J10.00 | 487 |  | ✓ |  | ✓ |
|  | Influenza due to other identified influenza virus with the same other identified influenza virus pneumonia | J10.01 | 487 |  | ✓ |  | ✓ |
|  | Influenza due to other identified influenza virus with other specified pneumonia | J10.08 | 487.0 and 488.11 |  | ✓ |  | ✓ |
|  | Influenza due to unidentified influenza virus with pneumonia | J11.0 | N/A |  | ✓ |  | ✓ |
|  | Influenza due to unidentified influenza virus with unspecified type of pneumonia | J11.00 | 487 |  | ✓ |  | ✓ |
|  | Influenza due to unidentified influenza virus with specified pneumonia | J11.08 | 487 |  | ✓ |  | ✓ |
|  | Influenza with pneumonia | N/A | 487^f^ |  | ✓ |  | ✓ |
| Other viral pneumonia | | J12^f^ | 480^f^ |  | ✓ |  | ✓ |
| **Bacterial and other pneumonia** | | | | | | | |
|  | *Streptococcus pneumoniae* pneumonia | J13 | 481 |  | ✓ |  | ✓ |
|  | *Hemophilus influenzae* pneumonia | J14 | 482.2 |  | ✓ |  | ✓ |
|  | Other bacterial pneumonia | J15^f^ | 482^f^ |  | ✓ |  | ✓ |
|  | Pneumonia due to other specified organism | J16^f^ | 483^f^ |  | ✓ |  | ✓ |
|  | Pneumonia in infectious diseases classified elsewhere | J17 | 484^f^ |  | ✓ |  | ✓ |
|  | Pneumonia, unspecified organism | J18^f^ | 486 |  | ✓ |  | ✓ |
| Influenza disease | | J09^f^, J10.1, J10.2, J10.8^f^, J11.1, J11.2, and J11.8^f^ | 488^f^ |  | ✓ |  | ✓ |
| Acute respiratory distress syndrome | | J80 | 518.82 |  | ✓ |  | ✓ |
| COPD^g^ with acute exacerbation | | J44.1 | 491.21 |  | ✓ |  | ✓ |
| Asthma acute exacerbation | | J45^f^ | 493^f^ |  | ✓ |  | ✓ |
| **Respiratory failure** | | | | | | | |
|  | Acute respiratory failure | J96.0^f^ | 518.81 |  | ✓ |  | ✓ |
|  | Acute and chronic respiratory failure | J96.2^f^ | 518.84 |  | ✓ |  | ✓ |
|  | Respiratory arrest | R09.2 | 799.1 |  | ✓ |  | ✓ |
| **Other acute lower respiratory tract infections** | | | | | | | |
|  | Acute bronchitis | J20^f^ | 466 |  | ✓ |  | ✓ |
|  | Acute bronchiolitis | J21^f^ | 466.1^f^ |  | ✓ |  | ✓ |
|  | Unspecified acute lower respiratory tract infection | J22 | 519.8 |  | ✓ |  | ✓ |
|  | Bronchitis, not specified as acute or chronic | J40 | 490 |  | ✓ |  | ✓ |
|  | Other COPD | J44 | N/A |  | ✓ |  | ✓ |
|  | COPD with acute lower respiratory tract infection | J44.0 | 491.22 |  | ✓ |  | ✓ |
|  | COPD, unspecified | J44.9 | 491.20 and 496 |  | ✓ |  | ✓ |
|  | Simple and mucopurulent chronic bronchitis | J41^f^ | 491^f^ |  | ✓ |  | ✓ |
|  | Unspecified chronic bronchitis | J42 | 491.9 |  | ✓ |  | ✓ |
|  | Emphysema | J43^f^ | 492^f^ |  | ✓ |  | ✓ |
|  | Bronchiectasis | J47^f^ | 494^f^ |  | ✓ |  | ✓ |
|  | Abscess of lung and mediastinum | J85 | 513 |  | ✓ |  | ✓ |
|  | Gangrene and necrosis of lung | J85.0 | N/A |  | ✓ |  | ✓ |
|  | Abscess of lung without pneumonia | J85.2 | 513 |  | ✓ |  | ✓ |
|  | Abscess of mediastinum | J85.3 | 513.1 |  | ✓ |  | ✓ |
|  | Abscess of lung with pneumonia | J85.1 | 513 |  | ✓ |  | ✓ |
|  | Pyothorax | J86^f^ | 510^f^ |  | ✓ |  | ✓ |
| Acute and chronic sinusitis | | J01^f^ and J32^f^ | 461^f^ and 473^f^ |  | ✓ |  | ✓ |
| Acute upper respiratory tract infections | | J00^f^ and J02^f^-J06^f^ | 460^f^ and 462^f^-465^f^ |  | ✓ |  | ✓ |
| **Signs and symptoms of ARI** | | | | | | | |
|  | Hemoptysis | R04.2 | 786.3 |  | ✓ | ✓ | ✓ |
|  | Cough | R05 | 786.2 |  | ✓ | ✓ | ✓ |
|  | Dyspnea, unspecified | R06.00 | 786.09 |  | ✓ | ✓ | ✓ |
|  | Shortness of breath | R06.02 | 786.05 |  | ✓ | ✓ | ✓ |
|  | Acute respiratory distress | R06.03 | N/A |  | ✓ | ✓ | ✓ |
|  | Stridor | R06.1 | 786.1 |  | ✓ | ✓ | ✓ |
|  | Wheezing | R06.2 | 786.07 |  | ✓ | ✓ | ✓ |
|  | Other abnormalities of breathing | R06.8 | N/A |  | ✓ | ✓ | ✓ |
|  | Apnea, NEC^h^ | R06.81 | 786.03 |  | ✓ | ✓ | ✓ |
|  | Tachypnea, NEC | R06.82 | 786.06 |  | ✓ | ✓ | ✓ |
|  | Other abnormalities of breathing or other symptoms involving head and neck | R06.89 | 784.99 |  | ✓ | ✓ | ✓ |
|  | Other dyspnea and respiratory abnormality | N/A | 786.09 |  | ✓ | ✓ | ✓ |
|  | Other symptoms involving respiratory system and chest | N/A | 786.9 |  | ✓ | ✓ | ✓ |
|  | Chest pain on breathing or painful respiration | R07.1 | 786.52 |  | ✓ | ✓ | ✓ |
|  | Asphyxia and hypoxemia | R09.0^f^ | N/A |  | ✓ | ✓ | ✓ |
|  | Asphyxia | R09.01 | 799.01 |  | ✓ | ✓ | ✓ |
|  | Hypoxemia | R09.02 | 799.02 |  | ✓ | ✓ | ✓ |
|  | Pleurisy | R09.1 | 511 |  | ✓ | ✓ | ✓ |
|  | Respiratory arrest | R09.2 | 799.1 |  | ✓ | ✓ | ✓ |
|  | Abnormal sputum | R09.3 | 786.4 |  | ✓ | ✓ | ✓ |
|  | Other specified symptoms and signs involving the circulatory and respiratory systems | R09.8^f^ | 478.19, 784.91, and 786.7 |  | ✓ | ✓ | ✓ |
| **Signs and symptoms of acute febrile illness** | | | | | | | |
|  | Fever | R50^f^ | N/A |  | ✓ | ✓ | ✓ |
|  | Fever presenting with conditions classified elsewhere | R50.81 | 780.61 |  | ✓ | ✓ | ✓ |
|  | Fever, unspecified | R50.9 | 780.6 |  | ✓ | ✓ | ✓ |
|  | Chills (without fever) | R68.83 | 780.64 |  | ✓ | ✓ | ✓ |
| **Signs and symptoms of acute nonrespiratory illness** | | | | | | | |
|  | Diarrhea | R19.7 | 787.91 |  | ✓ |  |  |
|  | Disturbance of smell and taste | R43^f^ | N/A |  | ✓ |  |  |
|  | Unspecified disturbances of smell and taste | R43.9 | 781.1 and V41.5 |  | ✓ |  |  |
|  | Headache | R51^f^ | 784 |  | ✓ |  |  |
|  | Myalgia | M79.1^f^ | 729.1 |  | ✓ |  |  |
|  | Sepsis—symptoms and signs specifically associated with systemic inflammation and infection | R65^f^ | 785.52 |  | ✓ |  |  |
|  | Other malaise | R53.81 | 780.79 |  | ✓ |  |  |
|  | Other fatigue | R53.83 | 780.79 |  | ✓ |  |  |
|  | Shock, unspecified | R57.9 | 785.5 |  | ✓ |  |  |
|  | Debility, unspecified | N/A | 799.3 |  | ✓ |  |  |
|  | Altered level of consciousness or altered mental status | R41.82 and R40^f^ | 780.97 and 780.0^f^ |  | ✓ |  |  |
|  | Weakness | R53.1 | 780.79 |  | ✓ |  |  |
|  | Nausea and vomiting | R11^f^ | 787^f^ |  | ✓ |  |  |
|  | Rash and other nonspecific skin eruption | R21^f^ | 782.1 |  | ✓ |  |  |
|  | Abdominal pain | R10^f^ | 789^f^ |  | ✓ |  |  |

^a^VISION-CLI definition refers to CLI definition used in VISION network studies.

^b^ARI: acute respiratory illness.

^c^N/A: not applicable.

^d^Check marks indicate that codes were used for the CLI definition. Empty cells indicate that codes were not used for this definition of CLI.

^e^SARS: severe acute respiratory syndrome.

^f^Indicates that all downstream ICD-10 codes were used; for example, R11^f^ indicates that all codes from R11.01-R11.99 were used.

^g^COPD: chronic obstructive pulmonary disease.

^h^NEC: not elsewhere classified.
